# Long-term follow-up of individuals at risk of or who developed resignation syndrome in childhood, were granted residence permits and cared for within the Stockholm region: a register study

**DOI:** 10.1186/s12888-026-07830-7

**Published:** 2026-01-29

**Authors:** Kasra Zarei, Karl Sallin, Mathias Mattsson, Predrag Petrovic, Olle Lindevall, Anna Ohlis, Anna-Clara Hollander

**Affiliations:** 1 Department of Global Public Health, Karolinska Institutet, Solna, Sweden; 2 Centre for Research Ethics and Bioethics (CRB), Uppsala Universitet, Uppsala, Sweden; 3 Department of Clinical Neuroscience, Karolinska Institute, Solna, Sweden; 4 Child Neurology Unit, Astrid Lindgren Children’s Hospital, Karolinska University Hospital, Solna, Sweden; 5 Centre for Psychiatric Research (CPF), Department of Clinical Neuroscience, Karolinska Institute, Solna, Sweden; 6 Center for Cognitive and Computational Neuropsychiatry (CCNP), Department of Clinical Neuroscience, Karolinska Institute, Solna, Sweden; 7 Barn-Och Ungdomspsykiatri, Stockholms Läns Sjukvårdsområde (SLSO), Stockholm, Sweden

**Keywords:** Resignation syndrome

## Abstract

**Objective:**

To investigate long-term outcomes of individuals at risk of or who developed resignation syndrome (RS) in childhood and who received residence permits in Sweden.

**Methods:**

We followed individuals born 1988–2002, living in Stockholm, in healthcare registers until December 2016 (*N* = 5,226). The exposed group was defined as exhibiting potential symptoms of RS of varying severity in childhood (*n* = 107), and compared with their siblings, refugees, unaccompanied migrants, individuals who received child and adolescent mental health services (CAMHS) without RS, and Swedish-born individuals with Swedish-born parents. We estimated the cumulative incidence of psychiatric diagnoses until age 18. Outcomes included psychiatric care utilization, prescribed/purchased psychotropic medication in adulthood, and education attainment. We estimated crude and adjusted hazard ratios (aHR) using Cox proportional hazards models and crude and adjusted odds ratios using multivariable logistic regression models, with 95% confidence intervals, and adjusted for socioeconomic factors.

**Results:**

The exposed group not only had the risk of RS or developed RS in childhood but a large share was also diagnosed with anxiety/PTSD, depression, eating disorders and attempted suicide. At follow-up, psychiatric care utilization and psychotropic medication prescription rates did not differ significantly between the exposed group (as the reference) and Swedish-born individuals with Swedish-born parents, nor among refugees or unaccompanied migrants. Individuals who received CAMHS without RS, had a significantly higher risk of having been prescribed medication (aHR 2.41; CI:1.51, 3.83) and using outpatient psychiatric care (aHR 1.78; CI:1.15, 2.75). Siblings of the exposed group had a significantly lower risk of using outpatient psychiatric care (aHR 0.28; CI:0.12, 0.67). The exposed group was similar to all the comparison groups with regards to having finished high school at age 22 except for unaccompanied migrants and the sibling group who had a significantly lower odds of the outcome. In a sensitivity analysis including only individuals with fully developed RS symptoms (*n* = 53), other comparison groups had lower relative risks of using outpatient psychiatric care compared with individuals with fully developed RS symptoms (the reference group), including the Swedish-born general population group (aHR 0.57; CI:0.35, 0.93), refugees (aHR 0.43; CI: 0.26, 0.72) and unaccompanied migrants (aHR 0.54; CI: 0.33, 0.86) but people who used CAMHS during childhood had no difference in relative risk.

**Conclusions:**

Despite severe baseline morbidity, individuals at risk of or who developed RS did not altogether exhibit an increased risk of psychiatric care during follow-up and attained an educational level on par with individuals who had received CAMHS. However, individuals with fully developed RS symptoms had a higher risk of psychiatric care utilization compared to Swedish-born children with Swedish-born parents. As the findings reflect a heterogeneous group of children assessed to be at varying risks of developing RS rather than only confirmed, fully developed RS cases, further research on long-term outcomes of RS is needed particularly in larger, representative samples of RS cases including those not granted residency permits in Sweden.

**Clinical trial number:**

Not applicable.

**Supplementary Information:**

The online version contains supplementary material available at 10.1186/s12888-026-07830-7.

## Introduction

Resignation syndrome (RS), or Uppgivenhetssyndrom in Swedish, is a condition characterized by anxiety and depressive symptoms progressing into stupor and eventually unresponsiveness approximating a comatose state [1–3]. RS was described in asylum seeking minors in Sweden with the first case noted in 1998 [4]. It was included in the Swedish version of the International Classification of Diseases, the ICD-10-SE (F32.3 A), in 2014 [3], but is not recognized elsewhere. The total number of cases has been estimated to exceed one thousand [4] with an estimated peak prevalence of 424 cases in 2003–2005 [5]. Afflicted individuals originated mainly from former Soviet republics in Central Asia and former Yugoslavia. Some came from areas of armed conflict, and most were from ethnic or religious minorities including Uighurs, Romani, Yezidis, and Armenians [1, 6]. Currently in Sweden, there are very few cases reported to the national patient registry [4].

RS typically debuts in the context of a negative asylum decision with a swift or prolonged deterioration. Varying clinical characteristics have prompted symptom grading (e.g., prodromal, fully developed, remission, etc.) [1, 3]. Prodromal symptoms include anxiety, social withdrawal, and depression. At its most advanced stage, motor behavior or response to pain is lacking, and tube feeding is customary [1, 7–9]. In a study of 46 patients with RS, the age of onset was between 11 and 12 years old in both sexes [6].

The nosology of RS has been subject to much debate. Diagnoses recognized in the ICD or the Diagnostic and Statistical Manual of Mental Disorders (DSM), such as functional neurological symptom disorder (conversion disorder), have been proposed [1, 8]. Other suggested entities include apathy, functional or depressive catatonia, and traumatic withdrawal syndrome [1, 5]. Pervasive refusal syndrome (PRS) [9, 10], unrecognized in international classification systems [11] has also been put forward [12–14] although controversially [4, 15]. It was ascribed to refugees with medically unexplained unresponsiveness on Nauru [16] who however exhibited self-mutilation of a kind unknown to RS.

The etiology of RS has also been debated. Trauma and stress were early on assumed causal [12] and a neurobiological model involving autonomic dysfunction was proposed similar to PRS [10]. However, in recent years the epidemiological qualities of RS – cases surging, endemic to Sweden but nowhere else, and affecting some immigrant groups but sparing others – have inspired accounts endorsing sociocultural and local contextual factors, and their interaction. The illness narrative implying stress of migration to be causal, and the medicolegal practice whereby a residency permit was asserted necessary for recovery and therefore a crucial part of the recommended treatment, were suggested to have promoted functional symptoms [1] but also feigned illness [4, 5]. Also, culture-specific help seeking patterns have been recognized [4]. The suggestion has thus been made that RS is crucially dependent on contextual factors [1, 4, 5] comparable to other psychiatric and psychological conditions [17].

An improved understanding of short- and long-term clinical and sociodemographic characteristics and outcomes of individuals who previously had RS or were at risk of developing RS is important to inform health care professionals and other individuals who encounter children with symptoms of RS, but also to help partially describe the aftermath of the two decades long endemic of RS in Sweden. The aim of this study, using Swedish national registers, is to examine long-term outcomes in terms of psychiatric care utilization, prescribed and purchased psychotropic medication, specific psychiatric diagnoses, and education attainment in individuals who were at risk of developing RS or had RS symptoms in childhood in Stockholm, Sweden and who have been granted residence permits in Sweden and compare them with their siblings, other refugee children, unaccompanied migrant minors, people who used child and adolescent mental health services (CAMHS) during childhood for disorders other than RS, as well as the general population of Swedish-born individuals with Swedish-born parents.

## Methods

### Data sources

Data for this study was extracted from a linked-register research database (known as “Psychiatry Sweden”), which consists of national and local registers [18, 19]. Contact with health care in Sweden is recorded in local and national administrative registers covering the entire population, which can be linked with other comprehensive registers of the total population. Data were collected from the following registers: the national patient register [20], the register of the total population (for identification of all individual residents in Sweden) [21], the multi-generation register (to link children with parents) [22], the longitudinal integration database for health insurance and labour market studies (LISA; for income and education data) that holds data from the labour market, educational and social sectors (since 1990) [23], the longitudinal database for studies of the immigrants’ integration (STATIV) which contains registers of migration related issues since 1997 and has information on the individuals’ country of birth and date of permanent residence permit [19]. Primary care for mental health, and specialized psychiatric care, including regional register-data from the child and adolescent psychiatric clinics in Stockholm (the “Pastill”-database) was obtained from the Stockholm region VAL-databases [24]. Pastill, has data on everyone who seeks clinical care within CAMHS in Stockholm (including reason for seeking care, diagnosis and time of diagnosis, etc.). Prescribed medication was obtained from the prescribed drug registry available since July 2005 [25]. In Sweden, all migrants with a permanent or temporary residence permit (i.e., with the right to live and work in Sweden) are entitled to health care. Adult immigrants with other legal status, for instance asylum-seekers and undocumented migrants, are not entitled to the same universal health care but do have the right to subsidized health care for care that cannot be deferred [18]. The current study thus excludes asylum seekers and undocumented migrants.

### Design and study cohort

**Fig. 1:**
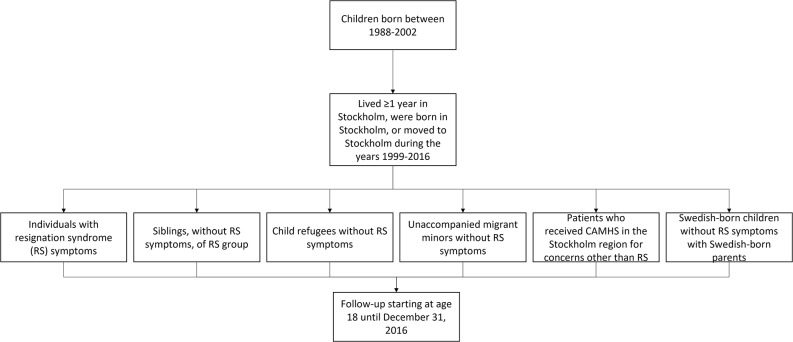
Flow-chart of study cohort design

The study population was an open cohort of children in Sweden with a permanent residence permit born between 1988 and 2002 who were born in Stockholm or moved to Stockholm before turning 18 (Fig. 1). The cumulative incidence of psychiatric disorders up until age 18 was determined for the study cohort, and then all individuals were followed up from their 18th birthday until they either received the outcomes (described below) or censorship due to leaving Stockholm, death or the study end-date (31 December 2016), whichever came first. It is possible that some minor healthcare utilization still occurred after censorship due to leaving Stockholm.

### Exposure and comparison groups

The exposure group was defined as children who, at baseline had been granted asylum in Sweden (and thus had been asylum seeking children before), and who had been determined to be at risk of developing RS based on an assessment by a physician at CAMHS in the Stockholm region sometime during the years 2005–2011 [3]. The clinical assessment was based on the MAsT evaluation scale [5], (an outline of the MAsT-scale is provided below). The MAsT evaluation was used among individuals in asylum seeking families exhibiting symptoms eliciting suspicion of RS and was not used for evaluation in the comparison groups. MAsT should not be perceived as a diagnostic instrument, but instead as a tool serving to illuminate the psychiatric and medical needs of children at risk of developing RS and to enable a common language used between different clinical disciplines . At grade 1 in the MAsT scale, the child is exhibiting obvious signs of depression and is at risk of entering a devitalized state. The child is passive, displays little interest in other people, and the child’s motor function is slow or characterized by uneasiness. The child’s appetite is poor; however, the child ingests sufficient food and fluids. The child performs also to some extent their daily routine, however with indifference and disinterest. At grade 2 in the MAsT scale, a child approaching a devitalized state makes poor contact, can nod in response or respond in one syllable, and possibly reacts to some events. The mobility is reduced, as the child has to be encouraged to move or be helped or supported when moving within or outside of the residence. Also, the appetite is reduced; the parents have to encourage the child to eat as they exhibit little interest in food or is devoid of hunger. The daily routine is maintained by the parents or at their request. At grade 3, the condition implies that the child is inapproachable, closes their eyes or looks at the floor, and displays little or no interest in the outside world. Even though the mobility is markedly reduced, the child lies down mostly and needs help to move. Ingestion requires a nasogastric tube or feeding by the parents. Furthermore, the child finds it difficult or close to impossible to perform a daily routine including personal hygiene and dressing; they may wet or soil themselves or they are mostly unaware of such bodily signals and can’t dress without the assistance of someone else [26]. Individuals who were diagnosed with grade 1 and 2 symptoms in the present sample did not develop more severe symptoms and have not been known to fully develop RS based on available data. However, they were still assessed as being at risk of developing RS by a physician at CAMHS, given the presence of risk factors including asylum seeking status. Although there was no ICD-diagnosis for RS ahead of 2014, individuals were still flagged as having RS symptoms within the CAMHS register (Pastill) during the years 2005–2011, which made it possible for us to include them in the present analysis.

The individuals with RS symptoms in childhood were compared with their siblings (who did not have RS symptoms in childhood). For additional comparisons, the cohort also included:

1. age/sex-matched child refugees [27]. For the creation of the refugee comparison group, children and young adults were categorized/defined as refugee minors if parents had been granted residence permit as refugees by the Swedish board of Migration, based on the United Nations refugee convention [28]. Some individuals lacked parental migration data but were defined as refugees if they themselves were registered as such.
2. unaccompanied migrant minors without RS symptoms. Among refugee children, there is a sub-group of unaccompanied refugee minors defined according to UNHCR [29] as children who have been separated from their parents, relatives, or other legal guardians. Individuals were defined as unaccompanied if they were registered as such in the STATIV database [18]. Among the unaccompanied children there were individuals with RS symptoms (< 5) and these individuals were removed from the unaccompanied minor group and added to the RS group.
3. age/sex-matched group consisting of patients without history of RS symptoms who received CAMHS in the Stockholm region (a ratio of 1 individual in the RS group to 10 matched individuals) [3]; and.
4. age/sex-matched group of Swedish-born children with Swedish-born parents (a ratio of 1 individual in the RS group to 10 matched individuals) independent if they had received CAMHS in the Stockholm region.

Comparisons were made between the exposed and the comparison groups separately as the comparison groups were not all mutually exclusive.

### Outcomes

Outcomes up until age 18 and during follow-up were determined using the diagnostic codes from the International Classification of Diseases, Tenth Revision (ICD-10), as well as the Diagnostic and Statistical Manual of Mental Disorders (DSM)-IV [30], Diagnostic Classification of Mental Health and Developmental Disorders of Infancy and Early Childhood (DC): 0–3 [31], and some specific to the CAMHS register when appropriate due to varying diagnostic coding practices among health workers. For simplicity in presentation, only the ICD-10 codes used are included below.

#### Outcomes up until age 18

We examined cumulative incidence of psychiatric care for all diagnoses except RS received in CAMHS up until 18 years including ADHD (ICD-10: F90-F90.9), anxiety including OCD and PTSD (ICD-10: F40-F43, F43.1), autism spectrum disorder (ASD, ICD-10: F84-F84.9), depression (ICD-10: F32-F33), eating disorders (ICD-10: F50-F50.9), intellectual disability (ICD-10: F70-F79), non-affective psychosis (ICD-10: F20-F29), or suicide attempt (ICD-10: T14.91), intentional self-harm or event of undetermined intent (ICD-10: X60-84, Y10-34). The time before age 18 summarizes cumulative incidence and does not take time into account and thus does not represent a true follow-up period.

#### Outcomes during follow-up (after age 18)

Outcomes during follow-up included any psychiatric care utilization (including primary care), specific diagnoses and prescribed medications during the follow-up period. Utilization of any outpatient mental health services was defined as at least one visit in specialized psychiatric outpatient care (ICD-10 codes F00-F99, X60-X84, Y10-Y34). Utilization of any inpatient mental health services was defined as at least one psychiatric inpatient treatment session based on the same ICD-10 codes. Additional outcomes included outpatient utilization (i.e., at least one visit in specialized psychiatric outpatient care) or inpatient utilization (i.e., at least one psychiatric inpatient treatment session) of condition-specific mental health services due to: ADHD (ICD-10: F90-F90.9), anxiety including PTSD (ICD-10: F40-F43, F43.1), autism spectrum disorder (ASD, ICD-10: F84-F84.9), depression (ICD-10: F32-F33), eating disorders (ICD-10: F50-F50.9), intellectual disability (ICD-10: F70-F79), non-affective psychosis (ICD-10: F20-F29), intentional self-harm (ICD-10: X60-84) or event of undetermined event (Y10-Y34).

Additional outcomes also included prescribed and purchased psychotropic medications, specifically prescription of: ADHD medications (ATC: N06BA01 to NO6BA04, N06BA09), antidepressants (ATC: N06A), antipsychotic drugs (ATC: N05A), anxiolytics (ATC: N05B), and sedative drugs (ATC: N05C), and a composite outcome defined as at least one prescription of any of the five medication categories.

Education outcomes included completion of secondary education and highest completed education (primary/elementary school, secondary school, and university education or missing) at the end of follow-up among those who had reached the age of 22 (*n* = 3561) at the end of follow-up.

### Demographic variables

Demographic variables included age, sex, geographic region of birth, highest level of parental education and highest level of parental income. Geographic area of origin was classified as “Central Asia” or “Other” given that most reported RS cases have been from Central Asia in other published studies, and there are not sufficient individuals with RS symptoms in childhood from other geographic areas or other countries in our dataset to allow for disaggregation given the need to preserve confidentiality [3]. Parent highest level of education was classified as “primary/elementary school”, “secondary school”, or “higher than secondary school”, and grouped into categories of “secondary school or lower” or “higher than secondary school” for modeling analyses.

To determine parental income, we determined the highest annual disposable family income throughout the follow-up period, categorized into quintiles in relation to the total population of each year, accounting for inflation. This variable is estimated by Statistics Sweden as an annual disposable income based on total family income (including welfare benefits), while weighting the total household income according to family composition and size, such that younger children are given lower weights than older household members and one adult is given a lower weight than two adults [18]. When an individual had parents with differing income values, the higher value was used to determine the highest parental income. For modeling analyses, the parental income quintiles were grouped into categories of the lower three income quintiles, and the higher two income quintiles.

### Statistical analyses

We used descriptive statistics to summarize cumulative incidence of psychiatric diagnosis except RS up to age 18 and psychiatric and educational outcomes within the six comparison groups during follow-up. Differences in outcome rates between exposed (*n* = 107) and the comparison groups; *n* = 5,119 were examined using chi-squared (χ²) tests and Fisher’s exact tests. We used multivariable logistic regression models and conditional logistic regression models expressed as adjusted odds ratios (AOR) with 95% confidence intervals (CI) for examining the association between study group and completion of secondary education at age ≥ 22 with the RS group as the reference. Conditional logistic regression models were used for the sibling comparison only. Cox proportional hazards models, under the proportional hazard assumption, were used for estimating the hazard ratios (HR) with 95% CI for first-contact outpatient psychiatric care and prescribed psychotropic medication in each study group compared to the RS group. For regression models except for the sibling comparison, we adjusted for demographics (sex, and when applicable, highest level of parental income categorized as the “bottom three income quintiles” vs. the “top two income quintiles”). For the sibling comparison, Cox proportional hazards models were used with corrections for standard error for clustering (clustered robust standard errors). A sensitivity analysis was also conducted running all the same models using only individuals with grade 3 symptoms based on the MAsT scale in the exposure group.

In the Cox regression models, age was used as an underlying timescale, such that everyone’s time-to-event was based on age at entry to age at event or censoring. Using age as the time scale allows for an analysis that shows how risk varies with age and has been suggested as less sensitive to biases compared to other timescales and therefore recommended for epidemiological cohort studies [32]. People leaving Stockholm or Sweden were censured at emigration. We used R (version 3.6) for data analysis and descriptive statistics and regression analyses, and STATA for data management and extraction.

### Ethical approval

This research has ethical approval from the Stockholm Regional Ethical Review Board (number 2010-1185-31-5) [18].

## Results

The study population included a total of 5,226 individuals. In total 107 individuals had been assessed at CAMHS to be an individual with RS symptoms in childhood (Table 1).

**Table 1:** Distributions of demographics and diagnoses among the study cohort at baseline (before age 18), n (%), by the RS population (RS) and the comparison groups: the siblings of the RS population (siblings), age/sex-matched accompanied child refugees (refugees), unaccompanied migrant minors without RS symptoms (unaccompanied), an age/sex-matched group consisting of patients without history of RS symptoms who received CAMHS in the Stockholm region (CAMHS), and an age/sex-matched group consisting of children with Swedish-born parents (Swedish born). The groups are not mutually exclusive.

Variable	RS	Siblings	Refugees	Unaccompanied	CAMHS	Swedish born
• Total	107	95	860	2029	1065	1070
• Sex						
Male	51 (47.7%)	53 (55.8%)	363 (42.2%)	1454 (71.7%)	508 (47.7%)	510 (47.7%)
Female	56 (52.3%)	42 (44.2%)	497 (57.8%)	575 (28.3%)	557 (52.3%)	560 (52.3%)
• Region of birth						
Central Asia	85 (79.4%)	54 (56.8%)	103 (12.0%)	655 (32.3%)	9 (0.8%)	0 (0.0%)
Other	22 (20.6%)	41 (43.2%)	757 (88.0%)	1374 (67.7%)	1056 (99.2%)	1070 (100.0%)
• Highest parental education						
Primary/Elementary school	11 (10.3%)	13 (13.7%)	148 (17.2%)	107 (5.3%)	58 (5.4%)	15 (1.4%)
Secondary school	32 (29.9%)	32 (33.7%)	196 (22.8%)	75 (3.7%)	403 (37.8%)	402 (37.6%)
> Secondary school	60 (56.1%)	49 (51.6%)	365 (42.4%)	101 (5.0%)	579 (54.4%)	653 (61.0%)
Missing	< 5	< 5	151 (17.6%)	1746 (86.1%)	25 (2.3%)	0 (0.0%)
• Highest parental income quintile						
1st (lowest)	16 (15.0%)	18 (18.9%)	264 (30.7%)	169 (8.3%)	14 (1.3%)	0 (0.0%)
2nd	23 (21.5%)	22 (23.2%)	129 (15.0%)	53 (2.6%)	27 (2.5%)	5 (0.5%)
3rd	15 (14.0%)	15 (15.8%)	103 (12.0%)	38 (1.9%)	41 (3.8%)	23 (2.1%)
4th	33 (30.8%)	26 (27.4%)	121 (14.1%)	24 (1.2%)	131 (12.3%)	102 (9.5%)
5th (highest)	16 (15.0%)	13 (13.7%)	100 (11.6%)	23 (1.1%)	830 (77.9%)	940 (87.9%)
Missing	< 5	< 5	143 (16.6%)	1722 (84.9%)	22 (2.1%)	0 (0.0%)
• Diagnosis before 18						
Attention-deficit/hyperactivity disorder	< 5	< 5	< 5	8 (0.4%)	166 (15.6%)	26 (2.4%)
Anxiety disorders (including PTSD)	89 (83.2%)	15 (15.8%)	43 (5.0%)	203 (10.0%)	371 (34.8%)	56 (5.2%)
Autism spectrum disorders	< 5	< 5	< 5	< 5	117 (11.0%)	23 (2.1%)
Depressive disorders	69 (64.5%)	6 (6.3%)	11 (1.3%)	83 (4.1%)	233 (21.9%)	44 (4.1%)
Eating disorders	39 (36.4%)	< 5	< 5	< 5	59 (5.5%)	10 (0.9%)
Intellectual disability	0 (0.0%)	0 (0.0%)	6 (0.7%)	6 (0.3%)	31 (2.9%)	< 5
Non-affective psychosis	< 5	0 (0.0%)	< 5	6 (0.3%)	6 (0.6%)	< 5
Suicide attempt or self-harm	26 (24.3%)	< 5	5 (0.6%)	21 (1.0%)	44 (4.1%)	< 5

There was a comparable number of females as males among the RS group (52.3% vs. 47.7%), the CAMHS group (52.3% vs. 47.7%), and the Swedish born group (52.3% vs. 47.7%). There were somewhat more males among the siblings of the RS group (55.8%) and more females in the refugee group (57.8%). There were few females among the unaccompanied migrant minors (28.3%). The majority of the RS group came from Central Asia (79.4%) whereas 56.8% of the sibling group and 32.2% of the unaccompanied migrant minors came from this region. In the RS group, 56.1% had at least one parent that had greater than a secondary school education, compared to 51.6% of the siblings, 42.4% of the refugees, 54.4% of the CAMHS group, and 61.0% of the Swedish born group (Table 1). The majority of the RS, sibling, and refugee groups belonged to the three lowest income quintiles, whereas nearly all of the CAMHS and Swedish born groups belonged to the highest two quintiles. Additional demographic characteristics are presented in Table 1.

### Baseline cumulative incidence of psychiatric diagnoses before age 18

The cumulative incidence until the 18th birthday of diagnoses among individuals with RS symptoms in childhood was 83.2% for anxiety disorders including PTSD, 24.3% suicide attempts, 64.5% for depression, and 36.4% for eating disorders (Table 1). Among individuals with RS symptoms in childhood, 53 (49.5%) had grade 3 symptoms, 16 (15.0%) had grade 2 symptoms, and 38 (35.5%) had grade 1 symptoms (Table 2). Distributions of region of birth and anxiety at baseline differed significantly by symptom grade (*p* < 0.05, Table 2) with those from Central Asia exhibiting more severe symptoms. The estimated mean age when individuals could have potentially had their first potential symptoms of RS was 11.7 years (standard deviation = 3.5 years), based on the last recorded visit to CAMHS.

**Table 2:** Distributions of demographics and diagnoses at baseline (before age 18), (n, %), in the RS- population and by grade (1–3), ranging from grade 1 (at risk of developing RS) to grade 3 (fully developed RS)

Variable	All	Grade 1	Grade 2	Grade 3
• Total	107	38	16	53
• Sex				
Male	51 (47.7%)	18 (47.4%)	9 (56.3%)	24 (45.3%)
Female	56 (52.3%)	20 (52.6%)	7 (43.8%)	29 (54.7%)
• Region of birth				
Central Asia	85 (79.4%)	23 (60.5%)	13 (81.3%)	49 (92.5%)
Other	22 (20.6%)	15 (39.5%)	< 5	< 5
• Highest parental education				
Primary/Elementary school	11 (10.3%)	5 (13.2%)	< 5	< 5
Secondary school	32 (29.9%)	8 (21.1%)	< 5	20 (37.7%)
> Secondary school	60 (56.1%)	22 (57.9%)	10 (62.5%)	28 (52.8%)
Missing	< 5	< 5	0 (0.0%)	< 5
• Highest parental income quintile				
1st (lowest)	16 (15.0%)	5 (13.2%)	< 5	10 (18.9%)
2nd	23 (21.5%)	7 (18.4%)	< 5	12 (22.6%)
3rd	15 (14.0%)	7 (18.4%)	< 5	6 (11.3%)
4th	33 (30.8%)	10 (26.3%)	6 (37.5%)	17 (32.1%)
5th (highest)	16 (15.0%)	6 (15.8%)	< 5	7 (13.2%)
Missing	< 5	< 5	0 (0.0%)	< 5
• Diagnosis before 18				
Attention-deficit/hyperactivity disorder	< 5	0 (0.0%)	0 (0.0%)	< 5
Anxiety disorders (including PTSD)	89 (83.2%)	27 (71.1%)	14 (87.5%)	48 (90.6%)
Autism spectrum disorders	< 5	0 (0.0%)	0 (0.0%)	< 5
Depressive disorders	69 (64.5%)	22 (57.9%)	14 (87.5%)	33 (62.3%)
Eating disorders	39 (36.4%)	6 (15.8%)	6 (37.5%)	27 (50.9%)
Intellectual disability	0 (0.0%)	0 (0.0%)	0 (0.0%)	0 (0.0%)
Non-affective psychosis	< 5	0 (0.0%)	0 (0.0%)	< 5
Suicide attempt or self-harm	26 (24.3%)	6 (15.8%)	< 5	18 (34.0%)

### Outcomes during follow-up

The mean age at end of follow-up was 23.3 years for individuals with RS symptoms in childhood; 19.0 years for their siblings; 23.4 years for other refugees; 23.6 years for unaccompanied migrant minors; 23.3 years for people who used CAMHS during childhood for other symptoms than RS; and 23.3 years for the Swedish-born general population (Table 3). At end of follow-up less than five had died in most groups and due to the low numbers, we will not report the exact numbers. The causes of death were often suicide and among individual with RS symptoms in childhood, all had died by suicide or death by unknown intent. Among individuals with RS symptoms in childhood, 63 (58.9%) attained secondary school education or higher (Table 3). The number of individuals with RS symptoms in childhood who were prescribed and had purchased at least one prescription of psychotropic medications at the end of follow-up < 5 for ADHD medications; 11 (10.3%) for antidepressants; < 5 for antipsychotics; 9 (8.4%) for anxiolytics; 12 (12.2%) for sedatives/hypnotics; and 20 (18.7%) for any of the medications (Table 3). During the follow-up period, 26 (25.3%) individuals with RS symptoms in childhood had used outpatient psychiatric care and < 5 had used inpatient psychiatric care (Table 3).

**Table 3:** Distributions of study outcomes, age at end of follow up, deaths, level of education at the end of study, prescribed medication, psychiatric outpatient care, psychiatric inpatient care, psychiatric outpatient care by diagnoses and psychiatric inpatient care by diagnoses (n, %) at the end of follow-up by the RS group and the comparison groups: the siblings of the RS population (siblings), age/sex-matched accompanied child refugees (refugees), unaccompanied migrant minors without RS symptoms (unaccompanied), an age/sex-matched group consisting of patients without history of RS symptoms who received CAMHS in the Stockholm region (CAMHS) and an age/sex-matched group consisting of children with Swedish-born parents (Swedish born). The groups are not mutually exclusive

Variable	RS	Siblings	Refugees	Unaccompanied	CAMHS	Swedish born
• Total	107	95	860	2029	1065	1070
• Age (mean years, SD, at end)	23.3 (3.5)	19.0 (6.9)	23.4 (3.7)	23.6 (3.9)	23.3 (3.5)	23.3 (3.5)
• Deaths	< 5	0	< 5	7 (0.3%)	< 5	< 5
• Level of education						
Primary/Elementary school	36 (33.6%)	26 (27.4%)	267 (31.0%)	874 (43.1%)	336 (31.5%)	219 (20.5%)
Secondary school	43 (40.2%)	22 (23.2%)	311 (36.2%)	724 (35.7%)	447 (42.0%)	458 (42.8%)
> Secondary school	20 (18.7%)	8 (8.4%)	172 (20.0%)	116 (5.7%)	187 (17.6%)	318 (29.7%)
Missing	8 (7.5%)	39 (41.1%)	110 (12.8%)	315 (15.5%)	95 (8.9%)	75 (7.0%)
• Prescribed medication						
ADHD	< 5	0 (0.0%)	< 5	15 (0.7%)	111 (10.4%)	31 (2.9%)
Antidepressants	11 (10.3%)	5 (5.3%)	73 (8.5%)	183 (9.0%)	276 (25.9%)	115 (10.7%)
Antipsychotics	< 5	0 (0.0%)	15 (1.7%)	51 (2.5%)	83 (7.8%)	21 (2.0%)
Anxiolytics	9 (8.4%)	< 5	84 (9.8%)	198 (9.8%)	219 (20.6%)	119 (11.1%)
Sedatives/Hypnotics	12 (11.2%)	< 5	52 (6.0%)	201 (9.9%)	187 (17.6%)	82 (7.7%)
• Any prescribed medication	20 (18.7%)	11 (11.6%)	165 (19.2%)	359 (17.7%)	333 (31.3%)	162 (15.1%)
• Any outpatient psychiatric care use	26 (24.3%)	6 (6.3%)	145 (16.9%)	455 (22.4%)	389 (36.5%)	229 (21.4%)
• Psychiatric outpatient care utilization						
0 visits	81 (75.7%)	89 (93.7%)	715 (83.1%)	1574 (77.6%)	676 (63.5%)	841 (78.6%)
1 visit	10 (9.3%)	< 5	46 (5.3%)	152 (7.5%)	62 (5.8%)	69 (6.4%)
2 visits	< 5	< 5	23 (2.7%)	66 (3.3%)	61 (5.7%)	29 (2.7%)
3 visits	< 5	0 (0.0%)	11 (1.3%)	37 (1.8%)	37 (3.5%)	17 (1.6%)
4 visits	0 (0.0%)	< 5	10 (1.2%)	29 (1.4%)	27 (2.5%)	14 (1.3%)
5 + visits	9 (8.4%)	0 (0.0%)	55 (6.4%)	171 (8.4%)	202 (19.0%)	100 (9.3%)
• Any inpatient psychiatric care use	< 5	< 5	24 (2.8%)	71 (3.5%)	111 (10.4%)	28 (2.6%)
• Inpatient psychiatric healthcare utilization						
0 visits	105 (98.1%)	94 (98.9%)	836 (97.2%)	1958 (96.5%)	954 (89.6%)	1042 (97.4%)
1 visit	0 (0.0%)	0 (0.0%)	13 (1.5%)	42 (2.1%)	47 (4.4%)	18 (1.7%)
2 visits	0 (0.0%)	< 5	< 5	10 (0.5%)	16 (1.5%)	7 (0.7%)
3 visits	< 5	0 (0.0%)	< 5	5 (0.2%)	14 (1.3%)	0 (0.0%)
4 visits	0 (0.0%)	0 (0.0%)	< 5	< 5	8 (0.8%)	0 (0.0%)
5 + visits	< 5	0 (0.0%)	< 5	11 (0.5%)	26 (2.4%)	< 5
• Any outpatient psychiatric care due to:						
Attention-deficit/hyperactivity disorder	< 5	0 (0.0%)	< 5	15 (0.7%)	134 (12.6%)	34 (3.2%)
Anxiety disorders (including PTSD)	18 (16.8%)	< 5	96 (11.2%)	253 (12.5%)	184 (17.3%)	143 (13.4%)
Autism spectrum disorders	0 (0.0%)	0 (0.0%)	5 (0.6%)	< 5	73 (6.9%)	20 (1.9%)
Depressive disorders	8 (7.5%)	< 5	48 (5.6%)	125 (6.2%)	138 (13.0%)	57 (5.3%)
Eating disorders	< 5	0 (0.0%)	8 (0.9%)	35 (1.7%)	28 (2.6%)	23 (2.1%)
Intellectual disability	0 (0.0%)	0 (0.0%)	14 (1.6%)	14 (0.7%)	17 (1.6%)	5 (0.5%)
Non-affective psychosis	0 (0.0%)	0 (0.0%)	< 5	22 (1.1%)	13 (1.2%)	5 (0.5%)
Suicide attempt or self-harm	< 5	0 (0.0%)	15 (1.7%)	46 (2.3%)	0 (0.0%)	20 (1.9%)
• Any inpatient psychiatric care use due to:						
Attention-deficit/hyperactivity disorder	0 (0.0%)	0 (0.0%)	< 5	< 5	18 (1.7%)	5 (0.5%)
Anxiety disorders (including PTSD)	< 5	0 (0.0%)	11 (1.3%)	16 (0.8%)	52 (4.9%)	5 (0.5%)
Autism spectrum disorders	0 (0.0%)	0 (0.0%)	< 5	< 5	14 (1.3%)	< 5
Depressive disorders	< 5	0 (0.0%)	< 5	9 (0.4%)	26 (2.4%)	10 (0.9%)
Eating disorders	< 5	0 (0.0%)	< 5	< 5	< 5	0 (0.0%)
Intellectual disability	0 (0.0%)	0 (0.0%)	< 5	< 5	< 5	0 (0.0%)
Non-affective psychosis	0 (0.0%)	0 (0.0%)	< 5	15 (0.7%)	6 (0.6%)	< 5
Suicide attempt or self-harm	0 (0.0%)	0 (0.0%)	0 (0.0%)	< 5	0 (0.0%)	0 (0.0%)

The proportion of individuals with RS symptoms in childhood who used any outpatient or inpatient psychiatric care were comparable to the general population, but lower than that of people who used CAMHS during childhood for symptoms other than concerns related to RS (Table 3). Outpatient psychiatric care use due to anxiety disorders (16.8%) and depressive disorders (7.5%) represented the primary reasons for psychiatric care use during follow-up among individuals with RS symptoms in childhood (Table 3). Compared to their siblings, the individuals with RS symptoms in childhood had significantly higher prevalence of outpatient psychiatric care due to anxiety and depression and had been prescribed more medication during follow-up (*p* < 0.05). We further describe distributions of the study outcomes by grade of RS symptoms in Table 4. There were essentially no significant differences between grade of RS symptoms, and sample size values in these comparisons are small.

**Table 4:** Distributions of study outcomes, age at end of follow up, deaths, level of education at the end of study, prescribed medication, psychiatric outpatient care, psychiatric inpatient care, psychiatric outpatient care by diagnoses and psychiatric inpatient care by diagnoses (n, %) at the end of follow-up in the RS group in total and by grade (1–3), ranging from grade 1 (at risk of developing RS) to grade 3 (fully developed RS)

Variable	All	Grade 1	Grade 2	Grade 3
• Total	107 (100.0%)	38 (100.0%)	16 (100.0%)	53 (100.0%)
• Age (mean years, SD, at end)	23.3 (3.5)	22.8 (3.9)	24.0 (2.6)	23.4 (3.3)
• Deaths	< 5	< 5	0 (0.0%)	< 5
• Level of education				
Primary/Elementary school	36 (33.6%)	14 (36.8%)	5 (31.3%)	17 (32.1%)
Secondary school	43 (40.2%)	16 (42.1%)	6 (37.5%)	21 (39.6%)
• > Secondary school	20 (18.7%)	< 5	5 (31.3%)	11 (20.8%)
• Missing	8 (7.5%)	< 5	0 (0.0%)	< 5
• Prescribed medication				
ADHD	< 5	0 (0.0%)	0 (0.0%)	< 5
Antidepressants	11 (10.3%)	< 5	< 5	6 (11.3%)
Antipsychotics	< 5	< 5	< 5	< 5
Anxiolytics	9 (8.4%)	< 5	< 5	< 5
Sedatives/Hypnotics	12 (11.2%)	5 (13.2%)	< 5	5 (9.4%)
• Any prescribed medication	20 (18.7%)	8 (21.1%)	< 5	9 (17.0%)
• Any outpatient psychiatric care use	26 (24.3%)	6 (15.8%)	< 5	18 (34.0%)
• Psychiatric outpatient care utilization				
0 visits	81 (75.7%)	32 (84.2%)	14 (87.5%)	35 (66.0%)
1 visit	10 (9.3%)	< 5	< 5	7 (13.2%)
2 visits	< 5	< 5	0 (0.0%)	< 5
3 visits	< 5	< 5	0 (0.0%)	< 5
4 visits	0 (0.0%)	0 (0.0%)	0 (0.0%)	0 (0.0%)
5 + visits	9 (8.4%)	< 5	< 5	6 (11.3%)
• Any inpatient psychiatric care use	< 5	< 5	< 5	0 (0.0%)
• Inpatient psychiatric healthcare utilization				
0 visits	105 (98.1%)	37 (97.4%)	15 (93.8%)	53 (100.0%)
1 visit	0 (0.0%)	0 (0.0%)	0 (0.0%)	0 (0.0%)
2 visits	0 (0.0%)	0 (0.0%)	0 (0.0%)	0 (0.0%)
3 visits	< 5	0 (0.0%)	< 5	0 (0.0%)
4 visits	0 (0.0%)	0 (0.0%)	0 (0.0%)	0 (0.0%)
5 + visits	< 5	< 5	0 (0.0%)	0 (0.0%)
• Any outpatient psychiatric care use due to:				
Attention-deficit/hyperactivity disorder	< 5	0 (0.0%)	0 (0.0%)	< 5
Anxiety disorders (including PTSD)	18 (16.8%)	< 5	< 5	12 (22.6%)
Autism spectrum disorders	0 (0.0%)	0 (0.0%)	0 (0.0%)	0 (0.0%)
Depressive disorders	8 (7.5%)	< 5	< 5	5 (9.4%)
Eating disorders	< 5	0 (0.0%)	0 (0.0%)	< 5
Intellectual disability	0 (0.0%)	0 (0.0%)	0 (0.0%)	0 (0.0%)
Non-affective psychosis	0 (0.0%)	0 (0.0%)	0 (0.0%)	0 (0.0%)
Suicide attempt or self-harm	< 5	< 5	0 (0.0%)	0 (0.0%)
• Any inpatient psychiatric care use due to:				
Attention-deficit/hyperactivity disorder	0 (0.0%)	0 (0.0%)	0 (0.0%)	0 (0.0%)
Anxiety disorders (including PTSD)	< 5	< 5	< 5	0 (0.0%)
Autism spectrum disorders	0 (0.0%)	0 (0.0%)	0 (0.0%)	0 (0.0%)
Depressive disorders	< 5	0 (0.0%)	< 5	0 (0.0%)
Eating disorders	< 5	< 5	0 (0.0%)	0 (0.0%)
Intellectual disability	0 (0.0%)	0 (0.0%)	0 (0.0%)	0 (0.0%)
Non-affective psychosis	0 (0.0%)	0 (0.0%)	0 (0.0%)	0 (0.0%)
Suicide attempt or self-harm	< 5	< 5	0 (0.0%)	0 (0.0%)

### Regression analysis of outcome during follow-up

Individuals with RS symptoms in childhood, representing the reference group, were compared with the other comparison groups (Tables 5 and 6). When compared with the Swedish-born population group and unaccompanied migrant minors, there were no statistically significant differences in relative risk in use of outpatient psychiatric care (Table 5). When compared with individuals with RS symptoms in childhood, siblings (HR = 0.28, 95%CI: 0.12, 0.67) and other refugees (HR = 0.65, 95%CI: 0.42, 1.00) had lower relative risk of outpatient psychiatric care (Tables 5 and 6). People who used CAMHS during childhood for disorders other than RS-related concerns had higher relative risk (HR = 1.78, 95%CI: 1.15, 2.75) of outpatient psychiatric care compared to individuals with RS symptoms in childhood (Table 5). For the outcome of any prescribed and purchased medication, there were no statistically significant differences compared to individuals with RS symptoms in childhood (Tables 5 and 6) except for individuals who used CAMHS during childhood for disorders other than RS who had a higher relative risk (HR = 2.41, 95%CI: 1.51, 3.83).

**Table 5:** Cox proportional-hazards models with age as underlying time scale and logistic regression models of the associations for the RS group (reference group) and Swedish born general population, CAMHS population, accompanied child refugees (refugees), and unaccompanied migrant minors without RS symptoms (unaccompanied). Separate models were performed for each comparison group. Adjusted models include sex and highest parental income quintile for comparisons to Swedish born, CAMHS, and refugees, and sex only for comparisons to unaccompanied migrant minors without RS symptoms

	Cox proportional-hazards model		Cox proportional-hazards model		Logistic regression model	
	Time any medication		Time first contact outpatient care		Finished high school at age > = 22	
Model	Crude	Adjusted	Crude	Adjusted	Crude	Adjusted
RS population	1	1	1	1	1	1
Swedish born	0.86 (0.55–1.36)	1.01 (0.56–2.06)	0.82 (0.54–1.23)	0.75 (0.45–1.24)	1.47 (0.98–2.20)	1.25 (0.71–2.17)
CAMHS	2.22 (1.43–3.44)*	2.41 (1.51–3.83)*	1.65 (1.11–2.47)*	1.78 (1.15–2.75)*	0.99 (0.66–1.48)	0.67 (0.42–1.06)
Refugees	0.70 (0.44–1.11)	0.70 (0.44–1.13)	0.59 (0.39–0.90)*	0.65 (0.42-1.00)*	0.91 (0.61–1.37)	1.16 (0.76–1.78)
Unaccompanied	0.84 (0.54–1.30)	0.84 (0.54–1.30)	0.81 (0.55–1.20)	0.79 (0.53–1.18)	0.65 (0.44–0.96)*	0.67 (0.45–0.99)*

*Denotes statistical significance (*p* < 0.05)

**Table 6:** Cox proportional-hazards model with age as underlying time scale and logistic regression model of the associations for the RS group (reference group) and siblings with correction for standard error for clustering, robust standard errors. Adjusted models include sex. Conditional logistic regression was used to model the completion of high school between the groups

	Cox regression with robust standard errors		Cox regression with robust standard errors		Conditional logistic regression model	
	Time any medication		Time first contact outpatient care		Finished high school at age > = 22	
Model	Crude	Adjusted	Crude	Adjusted	Crude	Adjusted
RS population	1	1	1	1	1	1
Siblings	0.64 (0.29–1.42)	0.64 (0.29–1.44)	0.27 (0.11–0.65)*	0.28 (0.12–0.67)*	0.40 (0.23–0.73)*	0.40 (0.22–0.73)*

Comparing the likelihood of completing secondary education at age ≥ 22, there were no statistically significant differences between individuals with RS symptoms in childhood, the Swedish-born general population group (AOR = 1.25, 95%CI: 0.71, 2.17; Table 5), people who used CAMHS during childhood for symptoms other than concerns related to RS (AOR = 0.67, 95%CI: 0.42, 1.06; Table 5), and other refugees (AOR = 1.16, 95%CI: 0.76, 1.78; Table 5). However, unaccompanied migrant minors (AOR = 0.67, 95%CI: 0.45, 0.99; Table 5) and siblings of individuals with RS symptoms in childhood (AOR = 0.40, 95%CI: 0.22, 0.73; Table 6) had lower likelihood of completing secondary education during follow-up compared to the individuals with RS symptoms in childhood. The results of the sensitivity analysis (Supplemental Tables 1–2) were partially consistent with the main analysis, with some notable differences. When compared with individuals with fully developed RS symptoms (grade 3) in childhood, other comparison groups had lower relative risks of using outpatient psychiatric care including the Swedish-born general population group (aHR 0.57; CI:0.35, 0.93) refugees (aHR 0.43; CI: 0.26, 0.72), and unaccompanied migrants (aHR 0.54; CI: 0.33, 0.86), but people who used CAMHS during childhood for symptoms other than concerns related to RS had no statistically significant difference in relative risk (HR = 1.26, 95%CI: 0.75, 2.11; Supplemental Table 1).

## Discussion and conclusion

The purpose of this study was to examine the long-term outcomes of individuals at risk of or who developed resignation syndrome in childhood with regards to utilization of psychiatric care (overall and for specific conditions), prescribed and purchased medication, and education-related outcomes, and also to compare the long-term outcomes with their siblings, other refugee children or children with an unaccompanied migrant minor background, children who received care at CAMHS without RS symptoms as well as Swedish-born children with Swedish-born parents.

The findings reflect a heterogeneous group of children, determined to be at risk of developing RS based on an assessment by a physician at CAMHS, rather than solely individuals with confirmed, fully developed RS. Despite severe morbidity at baseline, indicated by high cumulative incidence of diagnoses including anxiety (including PTSD), depression, and eating disorders, as well as suicide attempts, we found no indication of a higher utilization of psychiatric care - within the limitations of power and precision -among individuals with RS symptoms in childhood as compared with any of the groups during follow-up. In terms of educational outcomes, at age 22, individuals who had RS symptoms in childhood attained results not significantly different from that of the general population and superior to unaccompanied migrants and siblings. However, null differences may suggest, at least partially, uncertainty and opportunities for future investigation, rather than equivalence between compared groups. Furthermore, individuals with fully developed RS symptoms (grade 3) had a higher risk of outpatient psychiatric care utilization compared to the general population (Swedish-born children with Swedish-born parents), and similar risk of utilization compared to children who received care at CAMHS without RS symptoms. Additionally, the findings cannot be generalized to undocumented or denied-asylum RS populations, who may experience markedly different trajectories.

### The findings in context

The severe morbidity exhibited at baseline is in line with data from cohorts and reviews of RS [1, 2, 5, 6, 12–14, 26]. There are several possible explanations for the high cumulative incidence of anxiety, depression, and eating disorder (possibly failure to ingest) in the exposed group. As clinical entities, anxiety, depression and eating disorder are prodromal, or even symptoms of RS and the high cumulative incidences could reflect the fact that the group was developing RS or had RS. Alternatively, depression, anxiety, and failure to ingest could be premorbid or comorbid conditions to RS and serve as risk factors for one another, and RS, which could contribute to explaining their high cumulative incidences. Suicide-attempts are regardless of context related to depressive symptoms which are core to RS. In summary, the high cumulative incidences before age 18 corroborates the previous conception of a considerable psychiatric burden prior to or integral to the presentation of RS in childhood. Through reviewing medical records, further studies will have to try and assess whether the psychiatric morbidities at baseline were distinct from and preceded RS, were prodromes or comorbidities of RS, or RS mislabeled.

Although individuals with RS symptoms in childhood of varying severity had considerable psychiatric symptoms at baseline, we found - within the limitations of power and precision -no clear indication of a higher need overall of utilization of psychiatric care (overall and for specific conditions), prescribed and purchased medication, and difference in education-related outcomes at follow-up as compared to the general population. However, when focusing only on individuals with fully developed RS symptoms (MAsT grade 3), these individuals had a higher need of utilization of psychiatric care utilization – albeit with a small analytic sample size – compared to Swedish-born general population, and similar utilization compared to children who received care at CAMHS without RS symptoms.

There are multiple possible explanatory mechanisms for the findings – including clinical recovery, underutilization of services, resolution of social factors, or uncertainties in diagnostic labeling – which we discuss in greater detail. Such mechanisms could all contribute at least partially to the observed findings. Concerning clinical recovery, it could be that the exposed group received adequate care within CAMHS and was equipped with mental health promoting strategies, or related to underutilization of services, it could be the opposite; continued psychiatric symptom burdens, fears of stigma or an unsatisfactory experience of care within CAMHS could be discouraging in seeking future care despite needs, combined with other factors in immigrant populations that contribute to underutilization of health services. Both explanations could also be true to some extent. On the suggestions that RS is a functional or in some cases a feigned condition driven by social contexts [1, 8], the former category with broadly good prognoses in children and adolescents [33], and the latter fully reversible, no differences between the exposed group and the general population are predicted.

This data supports the notion that individuals with symptoms of RS can have good functional outcomes despite the severe morbidity at baseline, but those with fully developed RS symptoms may have a higher future need of utilization of psychiatric care utilization compared to the general population. Further research is needed assessing the finding that anxiety and depressive symptoms were more prevalent in the exposed group than in the general population. Concerning uncertainties in diagnostic labeling, while RS has mainly been a diagnosis applied to minors, in the transition from CAMHS to adult health care providers, different labelling practices could have resulted in some anxiety and depression diagnoses being given to individuals who developed RS, although this speculation needs to be assessed further as the diagnoses used for the primary outcome evaluated during follow-up in this study were assigned in the outpatient setting. The remaining incidence of anxiety and depression at follow-up could thus represent RS, or distinct or residual entities of anxiety and depressive disorders.

In the current study, we are unable to ascertain which, and in what order, psychiatric diagnoses occurred before RS symptoms during childhood, or if the diagnoses in fact were RS mislabeled. Starting follow-up at age 18 makes it less likely that symptoms of RS, which mostly have been reported to occur in childhood, were misclassified during follow-up. Evidence of comorbidities including anxiety and depression could provide insights into the etiology of RS. As mentioned, one hypothesis is that RS is a functional neurological disorder [8]. Risk factors for functional symptoms include anxiety and depression [34]. Symptoms of anxiety and depression are more common in individuals with functional symptoms compared to those without [35], and unless addressed, such symptoms may turn chronic. The combination of a severe baseline morbidity, including anxiety and depressive symptoms, and outcomes of no need for increased outpatient psychiatric care in some individuals, noted in this study, is compatible with the interpretation of RS as a functional disorder [1, 4, 5, 8]. Functional disorders in children and adolescents warrant psychosocial and psychiatric workup to eliminate underlying factors, including anxiety and depression, and require clear communication enabling the diagnoses to be accepted by the patient and family, and inadequate explanatory models to be abandoned. In such cases, a multidisciplinary team and biopsychosocial approach that includes neurologists, psychiatrists, psychologists, social workers and occupational therapists may be required [34].

The sibling comparison in this study was included to control variability in environmental, family/household factors and unmeasured confounders. There were significant differences between individuals in the RS group and their siblings in any outpatient psychiatric care, and outpatient care due to anxiety and depression. These differences warrant further investigation in a larger sample with longer follow-up to better evaluation and compare the health risks of experiencing RS within families.

Our findings in other immigrant comparison groups are consistent with the literature concerning immigrant psychiatric health and underutilization of health care services [18]. For instance, relative to the individuals with RS symptoms in childhood, siblings and other refugees had lower risk of using any outpatient psychiatric care.

### Limitations

There are notable limitations to this study. It is based on a limited number of individuals who were at risk of developing RS or who had fully developed RS in childhood [3], and not all have been confirmed to develop RS. For example, individuals who were only observed to have grade 1–2 symptoms (i.e., depressive symptoms) have, based on our data, not been known to fully develop RS. This non-specificity of prodromal symptoms could lead to potential misclassification in exposure status – i.e., it is possible that individuals in the RS group with stage 1–2 symptoms should have been included in one of the comparison groups such as unaccompanied migrant minors or refugee children that were only clinically evaluated at their healthcare visits per standard practice – which is significant given the already small number of individuals who had symptoms of RS in childhood in this study. Also, before 2014, there was no established diagnosis for children with RS, and like all other studies on RS using data from the time period, this study relied on clinical symptom descriptions [3]. With no validated assessment of symptoms available, clinicians may have rated the presence or absence of symptoms differently. This may have compromised exposure attribution with over or under-inclusion, and mislabeling could have in turn skewed the data. As a result, the exposed group in this study constitutes a heterogeneous group of children, determined to be at varying risks of developing RS based on an assessment by a physician at CAMHS.

Undocumented migrants and asylum-seekers – groups that historically have comprised many individuals with RS symptoms in childhood – with symptoms of RS or who fully developed RS (treated or not treated at CAMHS) were not included in the study and could potentially have markedly different health and educational outcomes due to not receiving needed services, and thus the results of this study cannot be generalized to them. The study of individuals with RS but without residence permits is difficult given the now minimal number of cases and the unknown location of previous and current cases. There are medical records from individuals with RS but not awarded residency to be assessed in another ongoing project. Retrospectively, some individuals and local non-governmental organizations have collected some data on patients – including cases of RS who were not allowed a residence permit – they provided services for [5, 6]. However, as they are not awarded personal identification numbers, these individuals are not included in national registers, which limits opportunities for epidemiological research.

All participants in this study have a permanent residence permit and were thus eligible to health care. Still, one caveat of this study is the underutilization of mental health care services previously observed in immigrants in Sweden [18, 19]. However, this limitation was accounted for by comparing exposed individuals to their siblings and refugee children and unaccompanied refugee children with similar patterns of underutilization.

With data on parental mental health limited in this group [18], we did not analyze the potential effects of parental mental illness on outcomes which would have been preferable considering importance to functional symptoms [35] and hypothesized precipitating and perpetuating effects on RS [5].

## Conclusion

As a result of these limitations, the results must be interpreted with caution. However, this study is one of the first and most complete studies on outcomes of individuals who were at risk of or developed RS and it contributes important knowledge. The debate surrounding RS has been politicized and polarized in Sweden [36] and different explanatory models have been proposed with little empirical evidence. This study provides empirical data which can be useful in a still ongoing debate about RS and its endemic properties [4]. RS is today essentially non-prevalent in Sweden, and to our knowledge very rare globally, but in the event new cases bearing resemblance to those of the past emerge, the results from this study could provide guidance for clinicians, patients and families. Although more studies are needed, conveying a potentially positive prognosis could serve to relieve stress and thereby contribute to recovery from RS at least as long as the hypothesis of a functional genesis is correct. Further work is needed to evaluate longer-term prognoses. Interviews and health surveys of individuals who fully developed RS both with and without residence permits granted, including undocumented individuals and asylum-seekers, could provide important knowledge which informs clinical guidance for RS or related symptoms including biopsychosocial, multidisciplinary treatment approaches and potential risk factors to address for preventive actions. 

## Electronic supplementary material

Below is the link to the electronic supplementary material.

Supplementary Material 1

## Data Availability

The data used in this study cannot be made publicly available according to Swedish data protection law. Any questions about the data can be addressed to the study authors.

## Abbreviations

RS: Resignation syndrome
PRS: Pervasive refusal syndrome
CAMHS: Child and adolescent mental health services
