# Supplementary material for: Long-term follow-up of individuals at risk of or who developed resignation syndrome in childhood, were granted residence permits and cared for within the Stockholm region: a register study
Source: BMC Psychiatry. 2026 Jan 29;26:160. doi: 10.1186/s12888-026-07830-7 (PMC12896039; doi:10.1186/s12888-026-07830-7)
Supplement: Supplementary file 1 — Supplementary Material 1 [file 12888_2026_7830_MOESM1_ESM.docx]

Supplemental Table 1: Sensitivity analysis of Cox proportional-hazards models with age as underlying time scale and logistic regression models of the associations for the RS group (grade 3 symptoms only: reference group) and Swedish born population, CAMHS population, accompanied child refugees (refugees), and unaccompanied migrant minors without RS symptoms (unaccompanied). Separate models were performed for each comparison group. Adjusted models include sex and highest parental income quintile for comparisons to Swedish born, CAMHS, and refugees, and sex only for comparisons to unaccompanied migrant minors without RS symptoms.

|  | Cox proportional-hazards model | | Cox proportional-hazards model | | Logistic regression model | |
| --- | --- | --- | --- | --- | --- | --- |
|  | Time any medication | | Time first contact outpatient care | | Finished high school at age >=22 | |
| **Model** | Crude | Adjusted | Crude | Adjusted | Crude | Adjusted |
| **RS population** | 1 | 1 | 1 | 1 | 1 | 1 |
| **Swedish born** | 0.95 (0.50-1.79) | 1.16 (0.52-2.62) | 0.56 (0.34-0.91)* | 0.57 (0.35-0.93)* | 1.28 (0.73-2.22) | 0.85 (0.38-1.81) |
| **CAMHS** | 2.40 (1.28-4.49)* | 2.60 (1.36-4.96)* | 1.13 (0.70-1.84) | 1.26 (0.75-2.11) | 0.86 (0.49-1.50) | 0.52 (0.27-0.97)* |
| **Refugees** | 0.77 (0.41-1.47) | 0.77 (0.40-1.49) | 0.40 (0.25-0.66)* | 0.43 (0.26-0.72)* | 0.80 (0.46-1.40) | 0.99 (0.55-1.78) |
| **Unaccompanied** | 1.00 (0.54-1.88) | 1.01 (0.54-1.90) | 0.55 (0.35-0.89)* | 0.54 (0.33-0.86)*^&^ | 0.56 (0.32-0.98)* | 0.59 (0.34-1.02) |

*Denotes statistical significance (*p*<0.05)

^&^Borderline proportional hazard assumption fulfilled

Supplemental Table 2: Sensitivity analysis of Cox proportional-hazards model with age as underlying time scale and logistic regression model of the associations for the RS group (grade 3 symptoms only: reference group) and siblings with correction for standard error for clustering, robust standard errors. Adjusted models include sex. Conditional logistic regression was used to model the completion of high school between the groups.

|  | Cox regression with robust standard errors | | Cox regression with robust standard errors | | Conditional logistic regression model | |
| --- | --- | --- | --- | --- | --- | --- |
|  | Time any medication | | Time first contact outpatient care | | Finished high school at age >=22 | |
| **Model** | Crude | Adjusted | Crude | Adjusted | Crude | Adjusted |
| **RS population** | 1 | 1 | 1 | 1 | 1 | 1 |
| **siblings** | 0.69 (0.32-1.48) | 0.73 (0.35-1.53) | 0.19 (0.09-0.40)* | 0.20 (0.10-0.41)* | 0.37 (0.14-1.00)* | 0.39 (0.14-1.05) |
